# Hydrostatic pressure: A very effective approach to significantly enhance critical current density in granular iron pnictide superconductors

**DOI:** 10.1038/srep08213

**Published:** 2015-02-03

**Authors:** Babar Shabbir, Xiaolin Wang, S. R. Ghorbani, Chandra Shekhar, Shixue Dou, O. N. Srivastava

**Affiliations:** 1 Institute for Superconducting and Electronic Materials, Australian Institute for Innovative Materials, University of Wollongong, North Wollongong, NSW 2522, Australia; 2 Department of Physics, Ferdowsi University of Mashhad, Mashhad, Iran; 3 Max Planck Institute for Chemical Physics of Solids, 01187 Dresden, Germany; 4 Department of Physics, Banaras Hindu University, Varanasi-221005, India

## Abstract

Pressure is well known to significantly raise the superconducting transition temperature, T_c_, in both iron pnictides and cuprate based superconductors. Little work has been done, however, on how pressure can affect the flux pinning and critical current density in the Fe-based superconductors. Here, we propose to use hydrostatic pressure to significantly enhance flux pinning and T_c_ in polycrystalline pnictide bulks. We have chosen Sr_4_V_2_O_6_Fe_2_As_2_ polycrystalline samples as a case study. We demonstrate that the hydrostatic pressure up to 1.2 GPa can not only significantly increase T_c_ from 15 K (underdoped) to 22 K, but also significantly enhance the irreversibility field, H_irr_, by a factor of 4 at 7 K, as well as the critical current density, J_c_, by up to 30 times at both low and high fields. It was found that pressure can induce more point defects, which are mainly responsible for the J_c_ enhancement. Our findings provide an effective method to significantly enhance T_c_, J_c_, H_irr_, and the upper critical field, H_c2_, for other families of Fe-based superconductors in the forms of wires/tapes, films, and single crystal and polycrystalline bulks.

Iron based superconductors have revealed wonderful superconducting properties, including high values of critical temperature (*T*_c_), critical current density (*J*_c_), upper critical field (*H*_c2_), and irreversibility field (*H*_irr_). They also exhibit low anisotropy and very strong pinning, which gives rise to high *J*_c_ (~10^6^ A/cm^2^) in both single crystals and thin films at both low and high fields^1^,^2^,^3^,^4^,^5^,^6^,^7^,^8^,^9^,^10^. The *J*_c_ and its field dependence in polycrystalline bulks and tapes/wires, however, are still lower than what is required for practical applications. Enhancement of *J*_c_ or flux pinning using various approaches has always been a main focus of research with a view to large current and high field applications. So far, three main methods have been used to increase the *J*_c_ in cuprates, MgB_2_, and iron based superconductors: 1) texturing processes to reduce the mismatch angle between adjacent grains and thus overcome the weak-link problem in layer-structured superconductors; 2) introducing point pinning centres by chemical doping and 3) high energy ion implantation or irradiation to introduce point defect pinning centres. *J*_c_ values achieved by the irradiation method have reached as high as 10^6^–10^7^ A/cm^2^ for both low and high fields in single crystals and thin films^11^,^12^,^13^. This method is not ideal, however, for *J*_c_ enhancement in polycrystalline pnictide superconductors.

As is well known, the weak-link issue is the predominant factor causing low *J*_c_, especially at high fields in pnictide polycrystalline samples, which must be overcome. In order to improve the *J*_c_ and its field dependence in granular superconductors, the following prerequisites should be met: i) strong grain connectivity; ii) introduction of more point defects inside grains; and iii) *T*_c_ enhancement, which can increase the effective superconducting volume as well as *H*_irr_ and *H*_c2_.

We have taken into account that the following facts relating to flux pinning mechanisms must be addressed before an effective method is introduced for polycrystalline pnictide superconductors. The coherence length is very short (ξ ≈ a few nm), so elimination of weakly linked grain boundaries is important to achieve high *J*_c_^14^. The nature of the pinning mechanism plays a vital role in *J*_c_ field dependence. It is noteworthy that a high pinning force can boost pinning strength and, in turn, leads to higher values of *J*_c_. The ideal size of defects for pinning should be comparable to the coherence length^15^. Therefore, point defect pinning is more favourable than surface pinning, as its pinning force is larger than for surface pinning at high field, according to the Dew-Hughes model^16^. Therefore, it is very desirable to induce more point defects in superconductors. Although chemical doping and high energy particle irradiation can effectively induce point defects and enhance *J*_c_ in high fields, *T*_c_ and low field *J*_c_ deteriorate greatly for various types of superconductors. Therefore, the ideal approach should be the one which can induce more point defects, with increased (or at least at no cost of) superconducting volume and *T*_c_, as well as strongly linked grain boundaries.

Hydrostatic pressure has been revealed to have a positive effect on *T*_c_ in cuprate and pnictide superconductors. For instance, high pressure of 150 kbars can raise *T*_c_ of Hg-1223 significantly from 135 K to a record high 153 K^17^. The *T*_c_ of hole doped (NdCeSr)CuO_4_ was increased from 24 to 33 K at 3 GPa by changing the apical Cu-O distance^18^. The enhancement of *T*_c_ for YBCO is more than 10 K at 2 GPa^19^. Excitingly, pressure also shows positive effects on *T*_c_ for various pnictide superconductors. Pressure can result in improvement of *T*_c_ from 28 to 43 K at 4 GPa for LaOFFeAs^20^. For Co doped NaFeAs, the maximum *T*_c_ can reach as high as 31 K from 16 K at 2.5 GPa^21^. Pressure can also enhance the *T*_c_ of La doped Ba-122 epitaxial films up to 30.3 K from 22.5 K, due to the reduction of electron scattering and increased carrier density caused by lattice shrinkage^22^. A huge enhancement of *T*_c_ from 13 to 27 K at 1.48 GPa was observed for FeSe, and it reached the high value of 37 K at 7 GPa^23^,^24^.

Beside the above-mentioned significant pressure effects on *T*_c_ enhancement, pressure can have more advantages that are relevant to the flux pinning compared to other methods. 1) It always reduces the lattice parameters and causes the shrinkage of unit cells, giving rise to the reduction of anisotropy. 2) Grain connectivity improvement should also be expected, as pressure can compress both grains and grain boundaries. 3) The existence or formation of point defects can be more favourable under pressure, since it is well known that the formation energy of point defects decreases with increasing pressure^25^,^26^,^27^. 4) Pressure can cause low-angle grain boundaries to migrate in polycrystalline bulk samples, resulting in the emergence of giant grains, sacrificing surface pinning thereafter. Hence, a higher ratio of point pinning centres to surface pinning centres is expected due to the formation energy and migration of grain boundaries under pressure. 5) The significant enhancement of *T*_c_, as above-mentioned, means that superconducting volumes should be increased greatly below or above the *T*_c_ without pressure. Moreover, the *H*_c2_, *H*_irr_, and *J*_c_ have to be enhanced along with the *T*_c_ enhancement. These are the motivations of our present study on the pressure effects on flux pinning and *J*_c_ enhancement in polycrystalline pnictide bulks. We anticipated that hydrostatic pressure would increase the superconducting volume, *H*_irr_, and *H*_c2_ due to *T*_c_ enhancement, increase the point defects, improve grain connectivity, and reduce the anisotropy in pnictide polycrystalline bulk samples.

There is some evidence for *J*_c_ enhancement under pressure in YBa_2_Cu_3_O_7−*x*_ (YBCO) single crystal, which emphasizes the pressure effects on transport *J*_c_ for different angle grain boundaries. A recent report also shows enhanced *J*_c_ in a pnictide single crystal which is free of grain boundaries^28^,^29^,^30^. As mentioned earlier, polycrystalline superconducting materials are commonly used in practical applications, as they are easy to fabricate at low cost as compared to single crystals/thin films. Their superconducting performance is hindered by grain boundaries, however, due to granularity. Therefore, it is more important to use an efficient approach to enhance the *J*_c_ in polycrystalline bulk samples. In this study, we chose a polycrystalline Sr_4_V_2_O_6_Fe_2_As_2_ sample to demonstrate the significant effects of the hydrostatic pressure on flux pinning and the significant enhancement of *J*_c_ and *T*_c_ in this granular sample. It has been reported that the *T*_c_ for this compound can range from 15–30 K, depending on fabrication process and carrier concentration^31^. Generally, Tc under pressure remains nearly constant (or little increase) for optimal doped superconductors and decreases linearly in the overdoped range. Under doped superconductors under pressure have dome-like plots for *T*_c_ vs. pressure, so we chose a Sr_4_V_2_O_6_Fe_2_As_2_ sample with the low *T*_c_ of 15 K for the proposed pressure effect investigation to ensure a clear pressure effect on *T*_c_^32^. Our results show that pressure can enhance the *J*_c_ by more than 30 times at 6 K and high fields in polycrystalline Sr_4_V_2_O_6_Fe_2_As_2_, along with *T*_c_ enhancement from 15 to 22 K at 1.2 GPa and *H*_irr_ enhancement by a factor of 4. Our analysis shows that pressure induced point defects inside the grains are mainly responsible for the flux pinning enhancement.

## Results and Discussion

**Figure 1:**
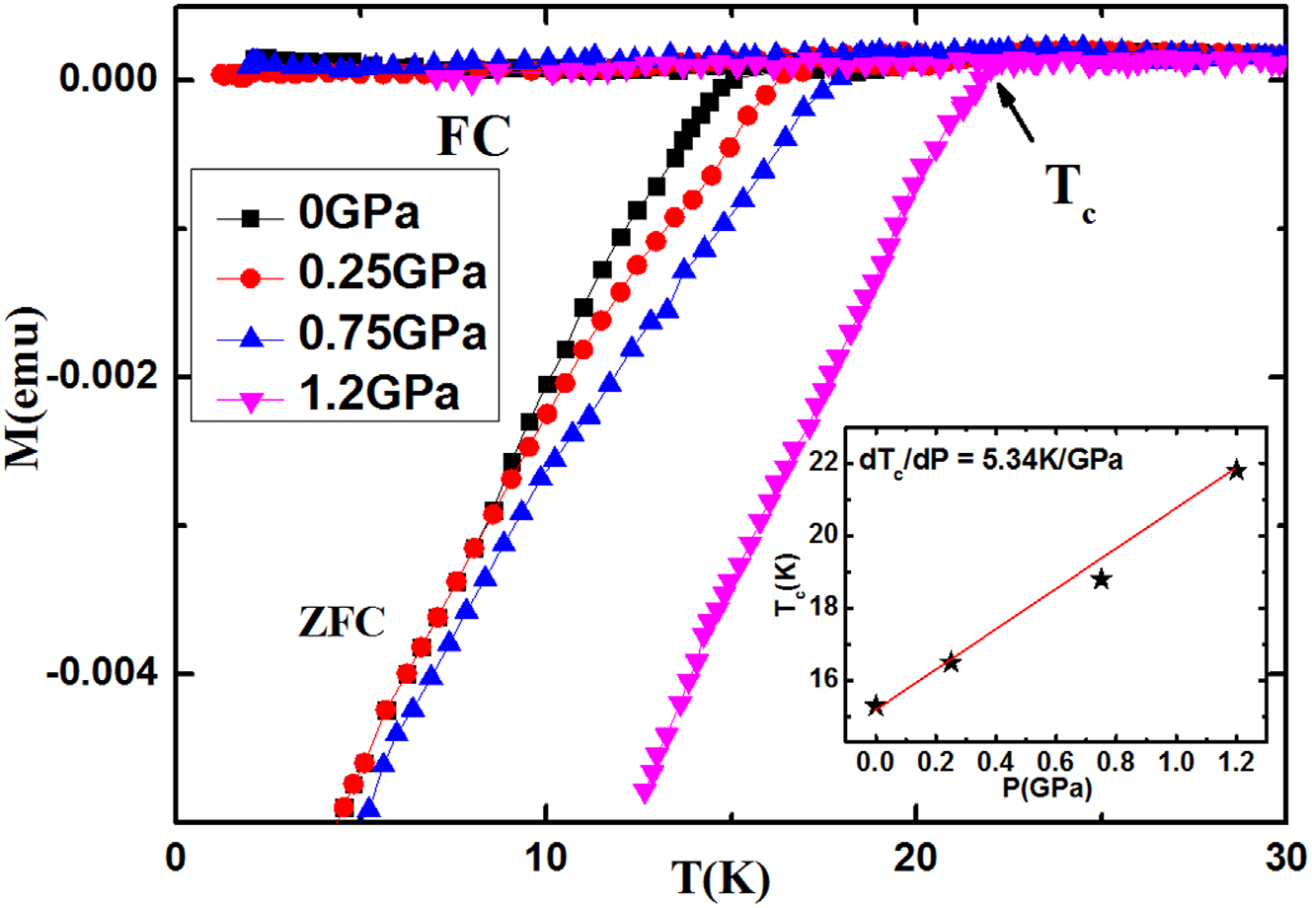
Temperature dependence of ZFC and FC moments at different pressures for Sr_4_V_2_O_6_Fe_2_As_2_. The inset shows the pressure dependence of T*_c_*.

Figure 1 shows the temperature dependence of the zero-field-cooled (ZFC) and field-cooled (FC) moments for Sr_4_V_2_O_6_Fe_2_As_2 _at different pressures. Pressure causes little change to the field-cooled branch, indicating that strong pinning is retained under pressure. The *T*_c_ without pressure is about 15 K, very similar to that of underdoped samples reported for Sr_4_V_2_O_6_Fe_2_As_2_ bulks^31^. Pressure enhances *T*_c_ linearly from 15.3 K for *P* = 0 GPa to 22 K for *P* = 1.2 GPa, with the pressure coefficient, *dT*_c_/*dP* = 5.34 K/GPa.

**Figure 2:**
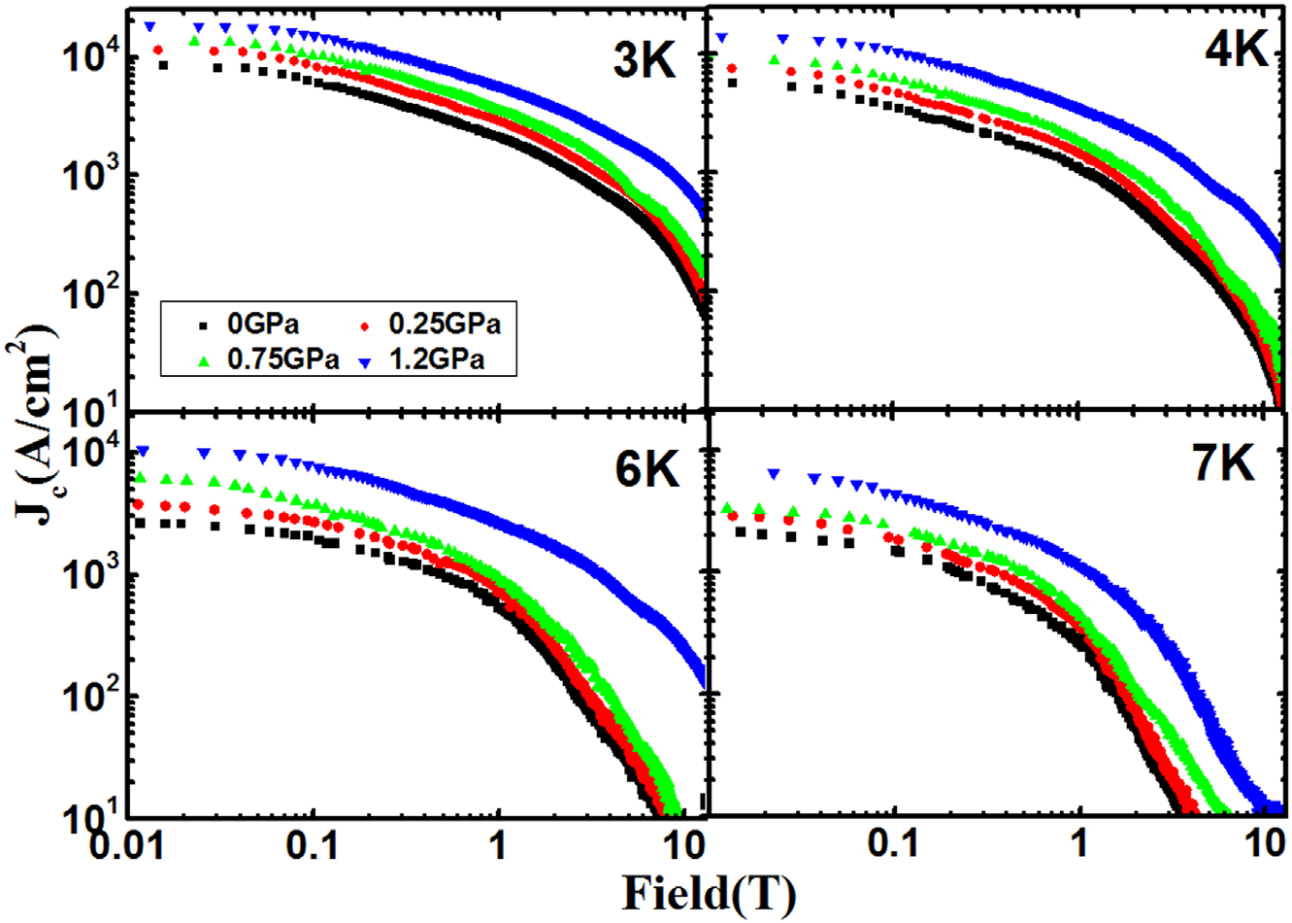
Field dependence of J*_c_* under different pressures at 3, 4, 6, and 7 K.

**Figure 3:**
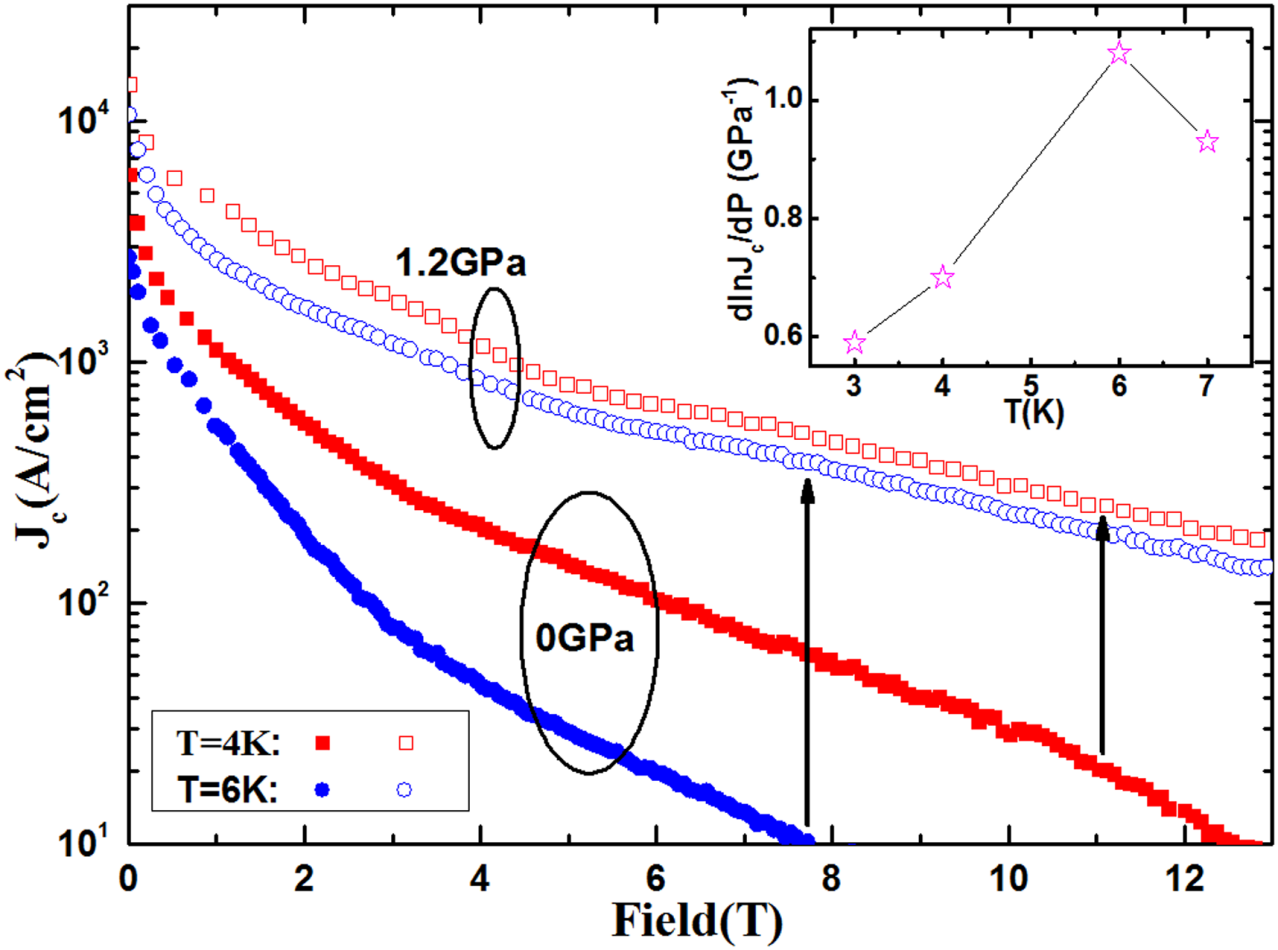
Comparison of J*_c_* at 0 and 1.2 GPa at 4 and 6 K. The inset shows d(lnJ*_c_*)/dP versus temperature, indicating enhancement of J*_c_* at a rate of 1.08 GPa^−1^ at zero field.

The *M*-*H* curves measured under different pressures indicate that the moment increases with increasing pressure. The field dependence of *J*_c _at different temperatures obtained from the *M*-*H* curves by using Bean's model under different pressures is shown in a double-logarithmic plot [i.e. Figure 2]. The remarkable effect of pressure towards the enhancement of *J*_c_ can be clearly seen. For *P* = 1.2 GPa, the *J*_c_ is significantly enhanced by more than one order of magnitude at high fields at 4 and 6 K, respectively, as shown in Figure 3.

**Figure 4:**
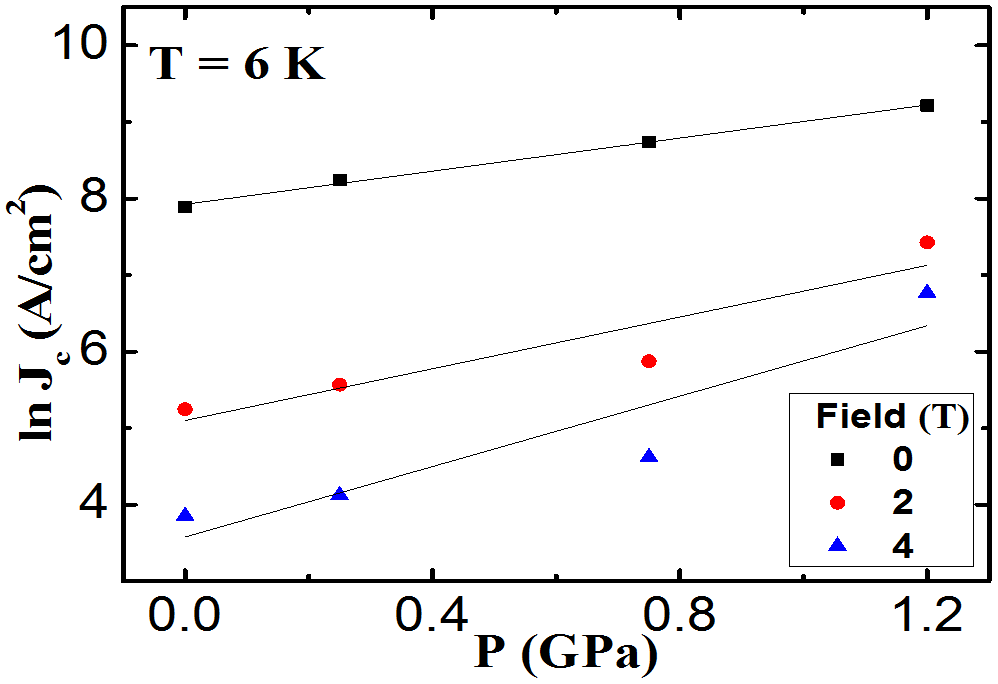
Pressure dependence of J*_c_* (logarithmic scale) at 0, 2, and 4 T at the temperature of 6 K.

The *J*_c_ at 6 K as a function of pressure at different fields is plotted in Figure 4. The solid lines in Figure 4 show linear fits to the data, which give the slopes (i.e. *d*(ln*J*_c_)/*dP*) of 1.09, 1.69, and 2.30 GPa^−1^ at 0, 2, and 4 T, respectively, indicating that the effects of pressure towards the enhancement of the *J*_c_ are more significant at high fields.

**Figure 5:**
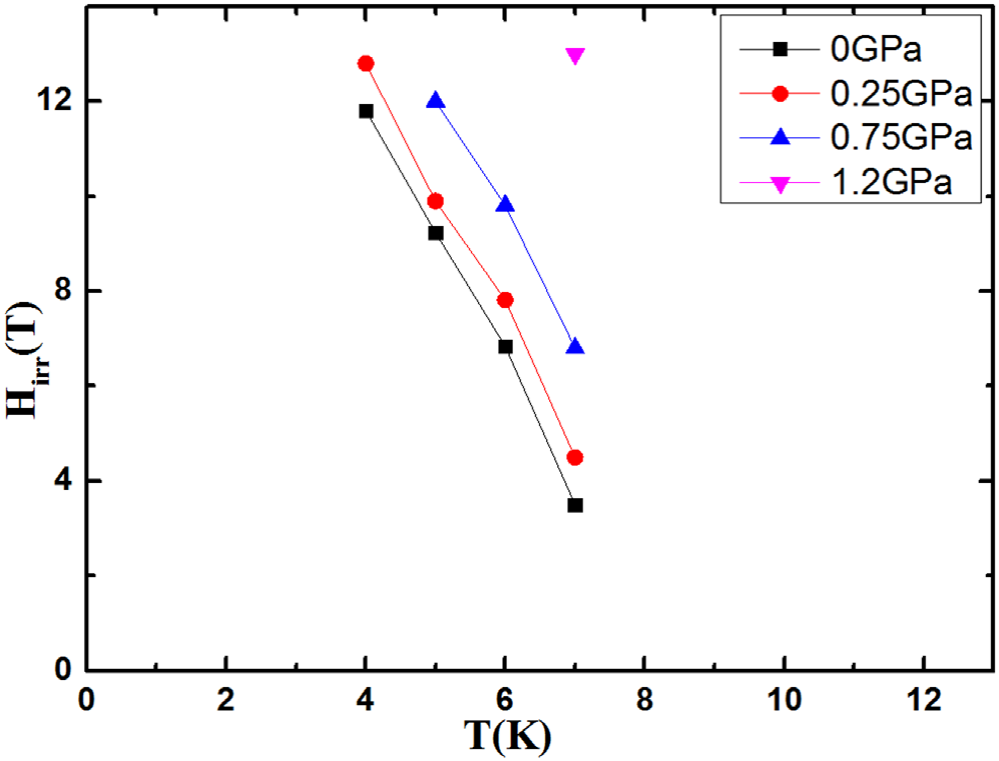
H*_irr_* vs. T for different pressures.

**Figure 6:**
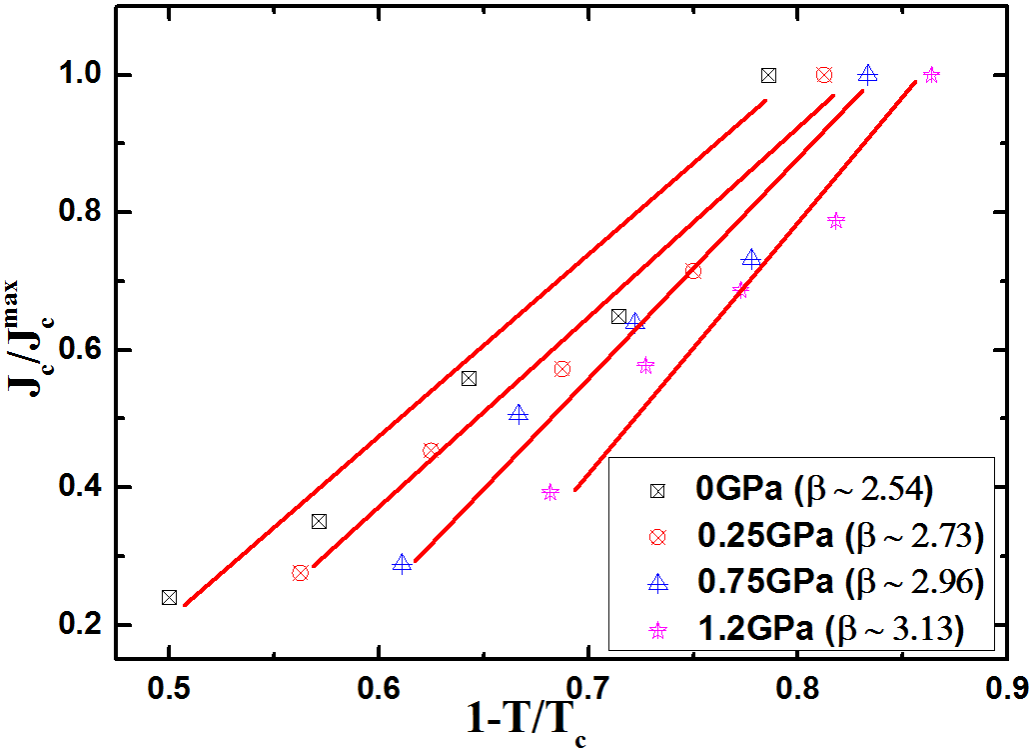
Logarithmic plot of J*_c_* as a function of reduced temperature at different pressures and fields.

We also found that the *H*_irr_ of Sr_4_V_2_O_6_Fe_2_As_2_ is greatly increased by pressure. The *H*_irr_ is defined as a field where *J*_c_ reaches as low as 10 A/cm^2^ in *J*_c_ vs field curves for different pressures and temperatures. As shown in Figure 5, the *H*_irr_ increases gradually with pressure and rises to 13 T from 3.5 T at 7 K. The *J*_c_ vs. reduced temperature (1-*T*/*T*_c_) at zero field and different pressures is plotted in Figure 6, which shows a rough scaling behaviour as *J_c_* ∝ (1−*T*/*T_c_*)*^β^* at different pressures. The slope of the fitting line, *β*, depends on the magnetic field. The exponent *β* (i.e. slope of the fitting line) is found to be 2.54, 2.73, 2.96, and 3.13 at 0, 0.25, 0.75, and 1.2 GPa, respectively. According to Ginzburg-Landau theory, the exponent “*β*” is used to identify different vortex pinning mechanisms at specific magnetic fields. It was found that *β* = 1 for non-interacting vortices, while β ≥ 1.5 indicates the core pinning mechanism^33^. The different values of β (i.e. 1.7, 2, and 2.5) were also reported for YBCO films which show the functioning of different core pinning mechanisms^34^,^35^. In addition, the exponent *β* values that we obtained are higher at higher pressures in our sample, indicating stronger improvement of *J*_c_ with temperature at high pressures.

For polycrystalline samples, high pressure can modify the grain boundaries through reducing the tunnelling barrier width and changing the tunnelling barrier height. The Wentzel-Kramers-Brillouin (WKB) approximation applied to a potential barrier gives the following simple expressions^36^: 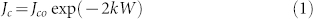
Where *W* is the barrier width, *k* = (2 mL)^1/2^/ħ is the decay constant, which depends on the barrier height *L*, ħ is the Planck constant, and *J*_c0_ is the critical current density for samples with no grain boundaries. The relative pressure dependence of *J*_c_ can be obtained from Eq. (1) as: 
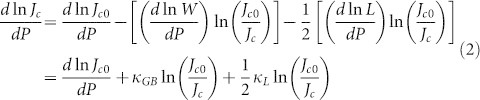
Where the compressibility in the width and height of the grain boundary are defined by *κ_GB_* = −*d* ln*W*/*dP* and *κ_L_* = −*d* ln*L*/*dP*, respectively. To estimate their contributions to the second and the third terms of Eq. (2) for *J*_c_ enhancement, we assume to a first approximation that *κ*_GB_ and *κ*_L_ are roughly comparable to the average linear compressibility values *κ*_a_ = -*d*ln*a*/*dP*(*κ*_GB_≈*κ*_a_) and *κ*_c_ = -*d*ln*c*/*dP*(*κ*_L_≈*κ*_c_) of Sr_4_V_2_O_6_Fe_2_As_2_ in the FeAs plane, where *a* and *c* are the in-plane and out-of-plane lattice parameters, respectively. We assume to a first approximation that *κ*_a_ = -*d*ln*a*/*dP* = −0.029 GPa^−1^, *κ*_c_ = -*d*ln*c*/*dP* = −0.065 GPa^−1^ 24, and the in-plane *J*_c0_≅ 10^5^ A/cm^2^ for a sample with no grain boundaries (single crystal)^37^. Setting *J*_c_≅ 2 × 10^3^ A/cm^2^ at the temperature of 6 K and ambient pressure, we find that (–*d*ln*W*/*dP*)ln(*J*_c0_/*J*_c_)≈ 0.11 and -0.5(*d*ln*L*/*dP*)ln(*J*_c0_/*J*_c_)]≈ 0.13, with both values adding up to 75% less than the above experimental value *d*ln*J*_c_/*dP* = 1.08 GPa^−1^. This result suggests that the origin of the significant increase in *J*_c_(T) under pressure does not arise from the compression of the grain boundaries. Therefore, Eq. (2) suggests that the main reason for the rapid increase of *J*_c_ with pressure is through point defects induced under pressure, i.e., *d*ln*J*_c0_/*dP* is responsible for approximately 75% of the total increase in the *J*_c_ with pressure.

**Figure 7:**
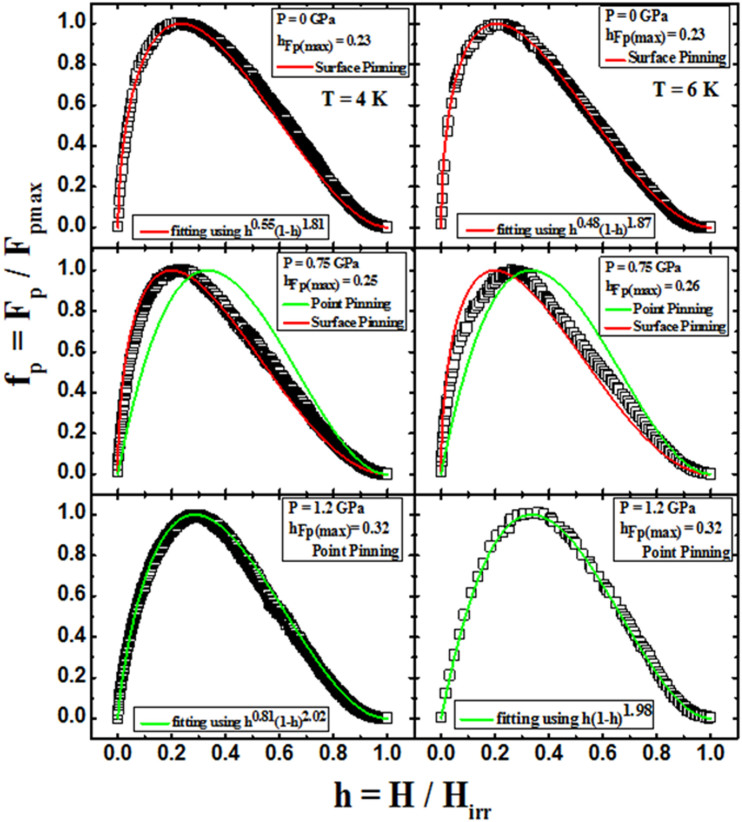
Plots of f*_p_* vs. H/H*_irr_* at different pressures (0, 0.75, and 1.2 GPa) for 4 (left) and 6 K (right) temperature curves. The experimental data is fitted through the Dew-Hughes model, and the parameters are shown.

In order to further understand the *J*_c_ enhancement under pressure, the pinning force *F*_p_ = *B* × *J*_c_ is calculated, and the scaling behaviour for the normalized pinning force *f*_p_ = *F*_p_/*F*_p,max_, is analysed for *h* = *H*/*H*_irr_. The results are shown in Figure 7 at 4 and 6 K under 0, 0.75, and 1.2 GPa. For the scaling, we can use the Dew-Hughes formula, i.e. *f_p_*(*h*) = *Ah^p^*(1−*h*)*^q^*, where *p* and *q* are parameters describing the pinning mechanism^16^. In this model, *p* = 1/2 and *q* = 2 describes surface pinning while *p* = 1 and *q* = 2 describes point pinning, as was predicted by Kramer^38^. At ambient pressure in the temperature range of 3–7 K, the best fits of the curves are obtained with *p* = 0.51 ± 0.03, *q* = 1.86 ± 0.03, which suggests that surface pinning is the dominant pinning mechanism in our sample. At 1.2 GPa, the best obtained values for *p* and *q* were 0.9 ± 0.1 and 2 ± 0.1, respectively, within the studied temperature range. This means that the dominant pinning mechanism is normal core point pinning for high pressures. Therefore, our results show that the pressure has induced a clear transformation from surface to point pinning.

Moreover, it is noteworthy that pressure can induce a reduction in anisotropy. The anisotropy is defined as *r* = *ξ_ab_*/*ξ_c_* where *ξ_ab_* is a coherence length along ab plane and *ξ_c_* along c plane. At high temperatures, the pressure dependence of *T*_c_, unit cell volume (*V*), and anisotropy (*r*) are interconnected through the following relation^39^; 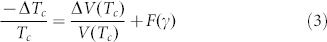
Where *F*(*γ*) = [*γ*(*P*)−*γ*(0)/*γ*(0)]. Although, no report for bulk modulus of Sr_4_V_2_O_6_Fe_2_As_2_ is yet available, we have tentatively used the bulk modulus (K = 62 GPa) of a similar superconductor i.e., SrFe_2_As_2_, to estimate Δ*V*(*T_c_*)/*V*(*T_c_*)), which is found to be ~ -0.016 at Δ*P* = 1 GPa, as it can be related to the bulk modulus as Δ*V*/*V* = -Δ*P*/*K*^40^. Our experimental results yield a value of 0.486 for Δ*T_c_*/*T_c_* = [*T_c_*(*P*)−*T_c_*(0)]/*T_c_*(0). By using these results, we can obtain *γ*(*P*) ≈ 0.53*γ*(0). Thus, we can conclude that the anisotropy has been reduced by almost half at high temperature by pressure. The decrease in the unit cell parameters suppresses its volume, leading to an increase in the Fermi vector *k*_F_ = (3*π*^2^*N*/*V*)^1/3^, where *N* is the total number of electrons in the system. The increase in the Fermi vector promotes enhancement of the coherence length along the *c*-axis (*ξ_c_* = ħ^2^*K_F_*/*πm*Δ where Δ is the uniform energy gap which, in turn, leads to the suppression of anisotropy.

In summary, hydrostatic pressure is a very effective means to significantly enhance *T*_c_, *J*_c_, *H*_irr_, and flux pinning in the granular pnictide superconductor Sr_4_V_2_O_6_Fe_2_As_2_. We demonstrate that the hydrostatic pressure can significantly increase *T*_c_ from 15 to 22 K, as well as increasing *J*_c_ by up to 30 times at both low and high field and increasing *H*_irr_ by a factor of 4 at P = 1.2 GPa. Pressure introduces more point defects inside grains, so that it is mainly responsible for *J*_c _enhancement. In addition, we found that the transformation from surface pinning to point pinning induced by pressure was accompanied by a reduction of anisotropy at high temperatures. Our findings provide an effective method to significantly enhance *T*_c_, *J*_c_, *H*_irr_, and *H*_c2_ for other families of Fe-based superconductors in the forms of wires/tapes, films, and single and polycrystalline bulks.

## Methods

For the polycrystalline Sr_4_V_2_O_6_Fe_2_As_2_ sample synthesis, the Fe (Alfa Aesar, 99.2%) and As (Alfe Aesar,99%) chips were sealed in evacuated quartz tube and heat treated for 12 hours at 700°C. Later, the stoichiometric amounts of V_2_O_5_*(*Aldrich, 99.6%*)* + ½ × SrO_2_*(*Aldrich*)* + 7*/*2 × Sr (Alfa Aesar, 99%*)* + 2 × FeAs were weighed, mixed, grounded thoroughly and palletized in rectangular form in a glove box in a high purity Ar atmosphere. The pellet was further wrapped in tantalum foil and then sealed in an evacuated (10−5 torr) quartz tube and put for heat treatments at 750 and 1150°C in a single step for 12 and 36 hours respectively. Finally, the quartz ampoule was allowed to cool naturally to room temperature.

The temperature dependence of the magnetic moments and the M-H loops at different temperatures and pressures were performed on Quantum Design Physical Property Measurement System (QD PPMS 14T) by using Vibrating Sample Magnetometer (VSM). We have used HMD High Pressure cell and Daphne 7373 oil as a pressure transmitting medium to apply hydrostatic pressure on a sample. The critical current density was calculated by using the Bean approximation.

## Author Contributions

X.L.W. conceived the pressure effects and designed the experiments. B.S. performed high pressure measurements and collected the data. C.S. and O.N.S. provided samples for this work. X.L.W., B.S., S.X.D., and R.G. contributed to the discussions and analysis of the data. X.L.W., B.S. and R.G. co-wrote the paper.
